# Functional redundancy of transcription factors explains why most binding targets of a transcription factor are not affected when the transcription factor is knocked out

**DOI:** 10.1186/1752-0509-9-S6-S2

**Published:** 2015-12-09

**Authors:** Wei-Sheng Wu, Fu-Jou Lai

**Affiliations:** 1 Department of Electrical Engineering, National Cheng Kung University, Tainan, Taiwan

## Abstract

**Background:**

Biologists are puzzled by the extremely low percentage (3%) of the binding targets of a yeast transcription factor (TF) affected when the TF is knocked out, a phenomenon observed by comparing the TF binding dataset and TF knockout effect dataset.

**Results:**

This study gives a plausible biological explanation of this counterintuitive phenomenon. Our analyses find that TFs with high functional redundancy show significantly lower percentage than do TFs with low functional redundancy. This suggests that functional redundancy may lead to one TF compensating for another, thus masking the TF knockout effect on the binding targets of the knocked-out TF. In addition, we show that seven classes of genes (lowly expressed genes, TATA box-less genes, genes containing a nucleosome-free region immediately upstream of the transcriptional start site (TSS), genes with low transcriptional plasticity, genes with a low number of bound TFs, genes with a low number of TFBSs, and genes with a short average distance of TFBSs to the TSS) are insensitive to the knockout of their promoter-binding TFs, providing clues for finding other biological explanations of the surprisingly low percentage of the binding targets of a TF affected when the TF is knocked out.

**Conclusions:**

This study shows that one property of TFs (functional redundancy) and seven properties of genes (expression level, TATA box, nucleosome, transcriptional plasticity, the number of bound TFs, the number of TFBSs, and the average distance of TFBSs to the TSS) may be useful for explaining a counterintuitive phenomenon: most binding targets of a yeast transcription factor are not affected when the transcription factor is knocked out.

## Background

The binding of transcription factors (TFs) to the promoters of their target genes is one of the most important mechanisms for transcriptional regulation of gene expression. Therefore, knowing the binding targets of TFs is helpful for understanding how cells respond to stimuli by regulating the gene expression repertoire. In 2004, Harbison et al. [1] performed ChIP-chip experiments to determine the binding targets of 203 yeast TFs in the rich media condition. Since then, many computational methods have used this TF binding dataset to reconstruct yeast transcriptional regulatory networks [2-5]. These methods are all based on one assumption: most, if not all, binding targets of a TF are regulated by this TF. This assumption is supported by three computational studies which estimate about 60% of the binding targets of a TF are indeed the regulatory targets of this TF [6-8].

However, this assumption was challenged by an experimental study conducted by Hu et al. in 2007 [9]. They performed microarray experiments to identify the differentially expressed genes in each of 263 TF knockout strains in the rich media condition. Then they compared the set of genes bound by a TF (retrieved from Harbison et al.'s study [1]) with the set of genes differentially expressed when this TF is knocked out (retrieved from their own study [9]). Surprisingly, they found that only 3% of the binding targets of a TF are affected by the knockout of this TF. That is, only 3% of the binding targets of a TF are indeed regulated by this TF.

Biologists are puzzled by this extremely low percentage and researchers have tried to explain this counterintuitive phenomenon. Several computational studies showed that by cleaning the noises in the TF binding dataset and applying advanced statistical analysis tools for the identification of differentially expressed genes in the TF knockout effect dataset, the percentage can only be improved to 6%, indicating that data analysis issue is not the main reason that causes this extremely low percentage [9-14]. Therefore, researchers started to find biological explanations for the low percentage. Two computational studies have shown that co-expression, protein sequence homology and shared protein-protein interactions may lead to one TF compensating for another, thus masking the TF knockout effect on the binding targets of the knocked-out TF [10,15]. In this study, our goal is to find out other plausible biological explanations for the surprisingly low percentage. Our analyses suggest that one TF property (functional redundancy) may lead to one TF compensating for another, thus masking the TF knockout effect on the binding targets of the knocked-out TF. In addition, we show that seven gene properties (low expression level, lacking a TATA box, containing a nucleosome-free region immediately upstream of the transcriptional start site (TSS), low transcriptional plasticity, a low number of bound TFs, a low number of TFBSs, and a short average distance of TFBSs to the TSS) are associated with a gene being insensitive to the knockout of its promoter-binding TFs.

## Methods

### TF binding dataset and TF knockout effect dataset

The TF binding dataset was downloaded from Harbison et al.'s study [1]. They performed ChIP-chip experiments to determine the significantly (determined by the p-value threshold) bound genes of 203 yeast TFs in the rich media condition. The TF knockout effect dataset was downloaded from Hu et al.'s study [9]. They performed microarray experiments to identify the significantly (determined by the p-value threshold) differentially expressed genes in each of 263 TF knockout strains grown in the rich media condition, the same growth condition used in the ChIP-chip experiments conducted by Harbison et al. [1]. A previous study showed that using the p-value threshold of 0.005 yields the highest overlap between the TF binding dataset and TF knockout effect dataset [10], so we adopted 0.005 as the p-value threshold in this study. Of the 203 TFs in the TF binding dataset, 173 were also in the TF knockout effect dataset. Therefore, the binding and knockout effect data of these 173 TFs were used in this study, which contained 11374 TF-gene binding relationships and 11986 TF-gene knockout effects among 173 TFs and 4065 genes. Then we compared the set of genes bound by a TF with the set of genes differentially expressed when this TF is knocked out. Similar to previous studies [9-14], on average only 4% (453/11374) of the TF-gene binding relationships had the TF-gene knockout effects. That is, only 4% of the TF binding dataset was overlapped with the TF knockout effect dataset.

### Calculation of the functional redundancy of each TF

The procedure of calculating the functional redundancy of TF *t *is as follows. First, calculate the functional similarity (*FS*) between TF *t *and TF *q *using the Jaccard similarity coefficient

$$FS\left(t,q\right)=\frac{\left|A_{t}\cap A_{q}\right|}{\left|A_{t}\cup A_{q}\right|},$$

where *A_t_*(or *A_q_*) is the set of functional annotation terms assigned to TF *t *(or TF *q*) according Gene Ontology database [16] and MIPS functional catalogue database [17] and |*A_t _*∩ *A_q_*| is the number of common functional annotation terms of TF *t *and TF *q*. Note that 0 ≤ *FS*(*t, q*) ≤ 1. *FS*(*t, q*) = 1 when TF *t *and TF *q *have completely the same set of functional annotation terms and *FS*(*t, q*) = 0 when TF *t *and TF *q *have completely different sets of functional annotation terms. Then the functional redundancy (*FR*) of TF *t *is defined as

(1) $$FR\left(t\right)=\underset{q}{\text{max}}FS\left(t,q\right).$$

Note that 0 ≤ *FR*(*t*) ≤ 1. TF *t *would have high functional redundancy if there exists another TF *q *whose functions are highly similar to the functions of TF *t*.

### Overlap percentage (OP) calculation

Following Gitter et al.'s approach [10], the percentage of the TF binding dataset that is overlapped with the TF knockout effect dataset for *M *(a set of TFs with some property, e.g. high functional redundancy) is calculated as

(2) $$OP=\frac{\underset{t\in M}{\sum }|G_{B}\left(t\right)\cap G_{K}\left(t\right)|}{\underset{t\in M}{\sum }|G_{B}\left(t\right)|}$$

where *G*_B_(t) is the set of genes significantly bound by TF *t, G_K_*(*t*) is the set of genes significantly affected by the knockout of TF *t*, and |G_B_(t)| is the number of genes significantly bound by TF *t*.

Similarly, the percentage of the TF binding dataset that is overlapped with the TF knockout effect dataset for *N *(a set of genes with some property, e.g. high expression levels) is calculated as

(3) $$OP=\frac{\underset{g\in N}{\sum }|T_{B}\left(g\right)\cap T_{K}\left(g\right)|}{\underset{g\in N}{\sum }|T_{B}\left(g\right)|}$$

where *T*_B_(g) is the set of TFs which significantly bind to gene g and *T*_K_(g) the set of TFs which significantly affect the expression of gene g when they are knocked out.

## Results and discussion

### The overlap percentage varies among different TFs and different genes

**Figure 1:**
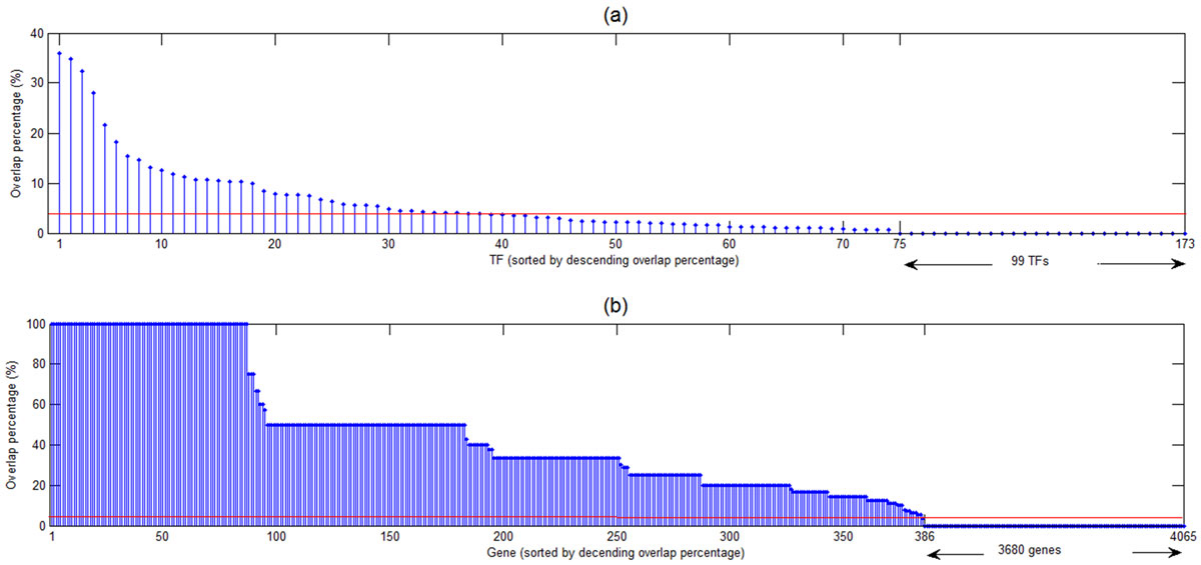
**The overlap percentage varies among different TFs and genes**. Although the overlap percentage for all 173 TFs (or all 4065 genes) under study is 4% (shown as the red line), the percentage actually varies among different TFs and different genes. (a) The overlap percentage for different TFs varies between 0% and 36%. (b) The overlap percentage for different genes varies between 0% and 100%.

Although on average only 4% of the TF binding dataset is overlapped with the TF knockout effect dataset, the percentage actually varies among different TFs and different genes. As shown in Figure 1, the percentage for different TFs varies between 0% and 36% and the percentage for different genes varies between 0% and 100% (see Additional file 1 for details). Identifying biological features that are associated with the overlap percentage may lead to biological explanations of the surprisingly low percentage of the binding targets of a TF affected when this TF is knocked out.

### Functional redundancy of TFs explains why most binding targets of a transcription factor are not affected when the transcription factor is knocked out

**Figure 2:**
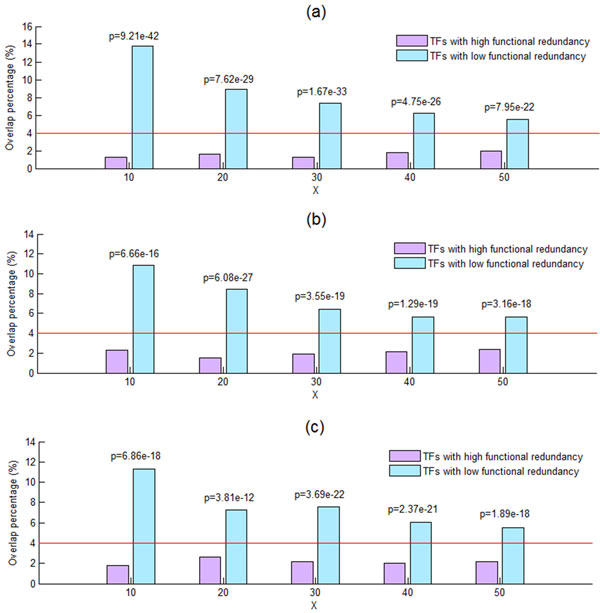
**Functional redundancy explains the low overlap percentage**. The set of TFs with high/low functional redundancy is defined as those TFs whose functional redundancy are among the top/bottom X% of the 173 TFs under study. By using the one-sided two-sample proportion test [28], we find that TFs with high functional redundancy show significantly (p-value << 0.001) lower overlap percentage than do TFs with low functional redundancy, suggesting that functional redundancy may explain why most binding targets of a TF are not affected when the TF is knocked out. Note that our result is robust against different choices (10, 20, 30, 40 or 50) of X and different sources ((a)MIPS [17], (b) GO:BP [16] or (c) GO:MF [16]) of functional annotation terms being used. The red line indicates the overlap percentage (4%) for all 173 TFs under study.

In order to test whether functional redundancy may lead to one TF compensating for another, thus masking the TF knockout effect on the binding targets of the knocked-out TF, let us define two sets of TFs. The first is the set of TFs with high functional redundancy, which is defined as those TFs whose functional redundancy calculated using Equation (1) are among the top X% (X = 10, 20, 30, 40 or 50) of the 173 TFs under study. The other is the set of TFs with low functional redundancy, which is defined as those TFs whose functional redundancy are among the bottom X% of the 173 TFs under study. As shown in Figure 2, TFs with high functional redundancy show significantly lower overlap percentage (calculated using Equation (2)) than do TFs with low functional redundancy, suggesting that functional redundancy may explain why most binding targets of a TF are not affected when the TF is knocked out. Note that our result is robust against different choices (10, 20, 30, 40 or 50) of X and different sources (MIPS or GO) of functional annotation terms being used.

### Lowly expressed genes have lower overlap percentage

**Figure 3:**
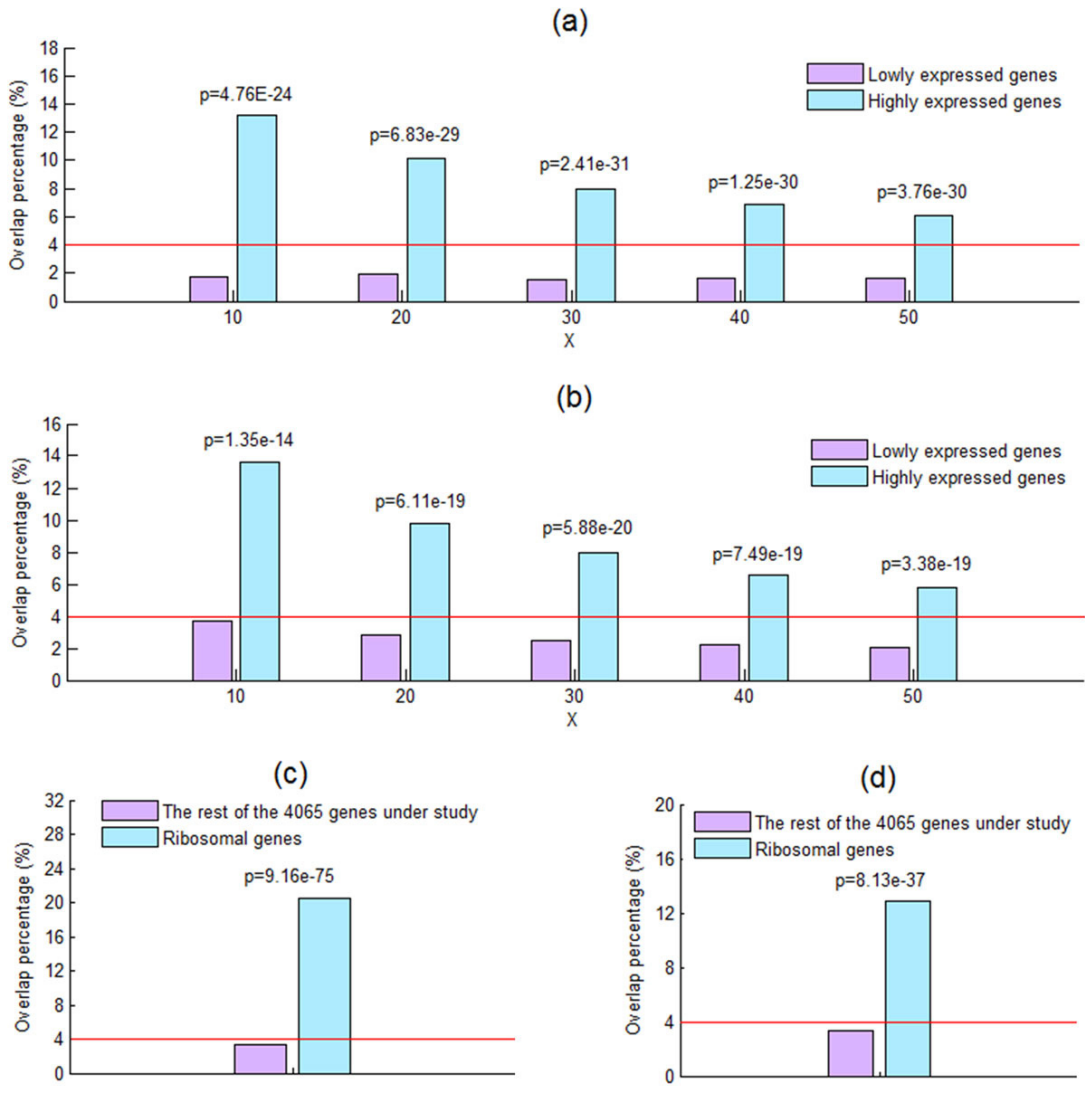
**High expression level and ribosomal genes are associated with high overlap percentage**. The set of lowly/highly expressed genes is defined as those genes whose expression levels are among the bottom/top X% of the 4065 genes under study. By using the one-sided two-sample proportion test [28], we find that lowly expressed genes show significantly (p-value << 0.001) lower overlap percentage compared with highly expressed genes, suggesting that low expression level is associated with a gene being insensitive to the knockout of its promoter-binding TFs. Note that our result is robust against different choices (10, 20, 30, 40 or 50) of X and different sources ((a) Holstege et al.'s study [18] or (b) Nagalakshmi et al.'s study [19]) of gene expression data being used. The red line indicates the overlap percentage (4%) for all 4065 genes under study. In addition, ribosomal genes show significantly (using the one-sided two-sample proportion test) higher overlap percentage compared with the rest of 4065 genes under study. This result further supports our finding that highly expressed genes show significantly higher overlap percentage. Note that our result is robust against different sources ((c) KEGG [20] or (d) MIPS [17]) of the list of ribosomal genes being used.

Since both ChIP-chip and TF knockout experiments were performed on the yeast cells grown in the rich media condition, we speculate that lowly expressed genes in the rich media condition have lower percentage of the TF binding dataset overlapped with the TF knockout effect dataset than do highly expressed genes. To test our speculation, let us define two sets of genes. The first is the set of lowly expressed genes, which is defined as those genes whose expression levels are among the bottom X% (X = 10, 20, 30, 40 or 50) of the 4065 genes under study. The other is the set of highly expressed genes, which is defined as those genes whose expression levels are among the top X% of the 4065 genes under study (see Additional file 2 for details). The gene expression data in the rich media condition was downloaded from Holstege et al.'s study [18] and Nagalakshmi et al.'s study [19]. As shown in Figures 3a and 3b, lowly expressed genes show significantly lower overlap percentage (calculated using Equation (3)) compared with highly expressed genes, suggesting that low expression level is associated with a gene being insensitive to the knockout of its promoter-binding TFs. Note that our result is robust against different choices (10, 20, 30, 40 or 50) of X and different sources (Holstege et al.'s study or Nagalakshmi et al.'s study) of gene expression data being used.

Ribosomal genes are known to be highly transcribed in the rich media condition. If our finding is biologically meaningful, we expect that ribosomal genes have higher overlap percentage compared with the rest of the 4065 genes under study. To test this assertion, we downloaded two lists of ribosomal genes from KEGG ribosome pathway: sce03010 [20] and MIPS functional category: 12.01.01 ribosomal proteins [17]. As expected, ribosomal genes show significantly higher overlap percentage (calculated using Equation (3)) compared with the rest of the 4065 genes under study (see Figures 3c and 3d), thus further strengthen our finding. Note that our result is robust against different sources (KEGG or MIPS) of the list of ribosomal genes being used.

### TATA box-less genes have lower overlap percentage

**Figure 4:**
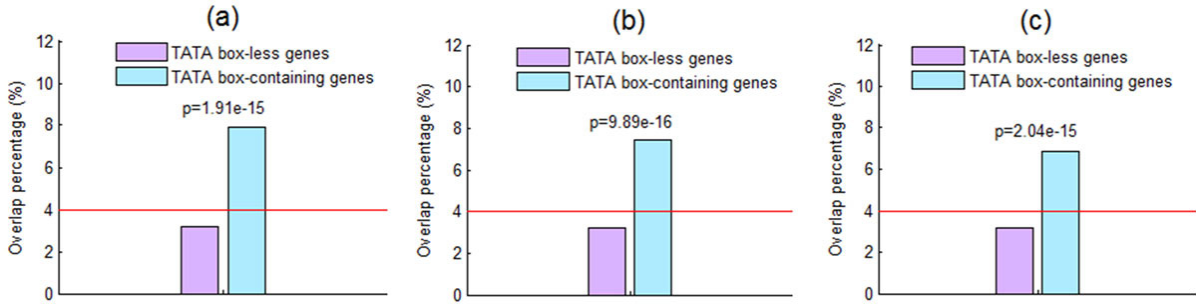
**Lacking a TATA box is associated with a gene being insensitive to the knockout of its promoter-binding TFs**. By using the one-sided two-sample proportion test [28], we find that TATA box-less genes show significantly (p-value << 0.001) lower overlap percentage compared with TATA box-containing genes, suggesting that lacking a TATA box is associated with a gene being insensitive to the knockout of its promoter-binding TFs. Note that our result is robust against three different criteria ((a), (b), and (c)) for defining TATA box-containing genes being used (see Basehoar et al.'s study [21] for more details). The red line indicates the overlap percentage (4%) for all 4065 genes under study.

It is known that TATA box-less and TATA box-containing genes are distinctly regulated [21]. TATA box-less genes tend to be housekeeping genes, have a sharply peaked TF binding site (TFBS) distribution and are constitutively expressed, while TATA box-containing genes are usually associated with environmental stress responses, dispersed TFBS distribution and variably expressed under different conditions [21-25]. It is interesting to know whether these two classes of genes differ in their overlap percentage. The lists of TATA box-less genes and TATA box-containing genes were downloaded from Basehoar et al.'s study [21]. Depending on how stringent the criterion for defining a TATA box is, three possible lists of TATA box-containing genes were defined by Basehoar et al. [21]. As shown in Figure 4, TATA box-less genes show significantly lower overlap percentage (calculated using Equation (3)) compared with TATA box-containing genes, suggesting that lacking a TATA box is associated with a gene being insensitive to the knockout of its promoter-binding TFs. Note that our result is robust against different criteria of defining TATA box-containing genes.

### Genes containing a nucleosome-free region (NFR) have lower overlap percentage

**Figure 5:**
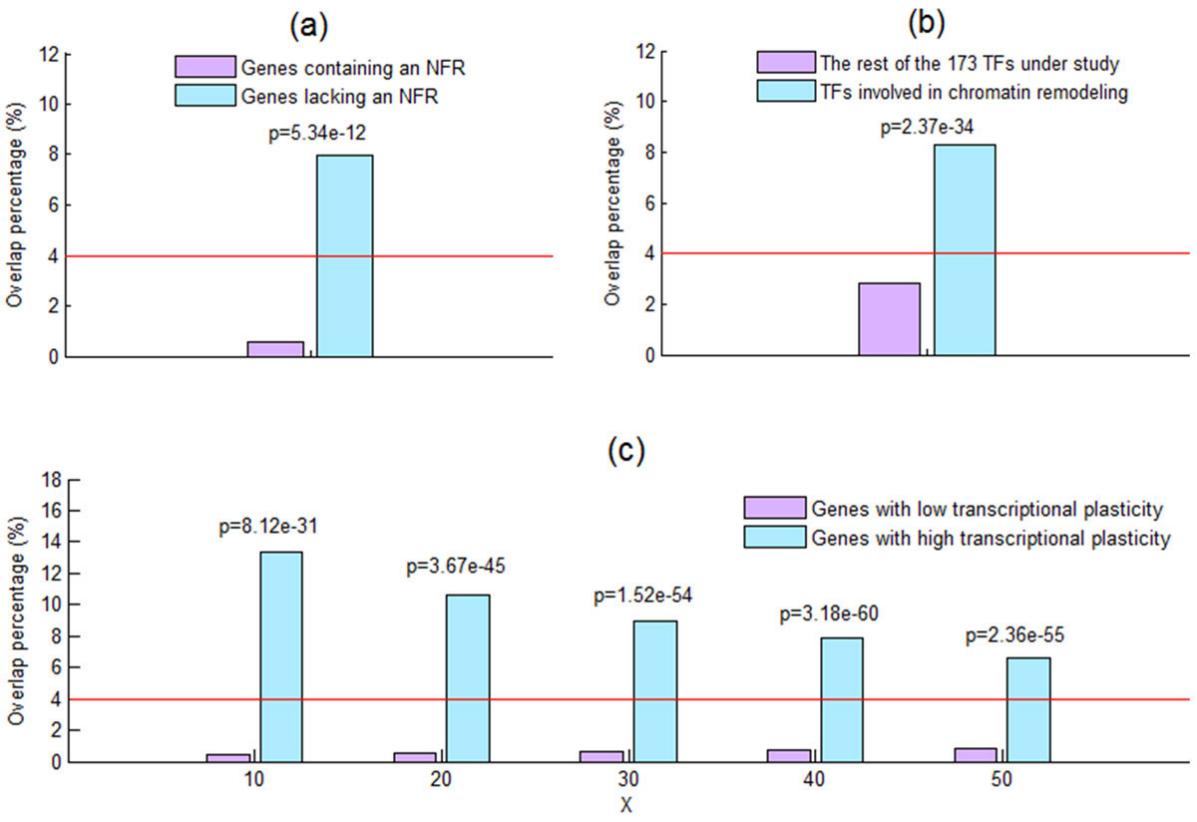
**NFR, chromatin remodelling TFs and transcriptional plasticity are associated with the overlap percentage**. (a) By using the one-sided two-sample proportion test [28], we find that genes containing an nucleosome free region (NFR) show significantly (p-value << 0.001) lower overlap percentage compared with genes lacking an NFR, suggesting that containing an NFR immediately upstream of the TSS is associated with a gene being insensitive to the knockout of its promoter-binding TFs. The red line indicates the overlap percentage (4%) for all 4065 genes under study. (b) TFs involved in chromatin remodelling show significantly (using the one-sided two-sample proportion test) higher overlap percentage compared with the rest of the 173 TFs under study. This result further supports our finding that genes lacking an NFR show significantly higher overlap percentage. (c) Low transcriptional plasticity is associated with a gene being insensitive to the knockout of its promoter-binding TFs. The set of genes with low/high transcriptional plasticity is defined as those genes whose transcriptional plasticity are among the bottom/top X% of the 4065 genes under study. By using the one-sided two-sample proportion test, we find that genes with low transcriptional plasticity show significantly (p-value << 0.001) lower overlap percentage than do genes with high transcriptional plasticity, suggesting that low transcriptional plasticity is associated with a gene being insensitive to the knockout of its promoter-binding TFs. Note that our result is robust against different choices (10, 20, 30, 40 or 50) of X being used.

In yeast, the capacity to modulate gene expression upon changing conditions (i.e., transcriptional plasticity) correlates with the organization of their promoter nucleosomes [26]. Genes containing an NFR immediately upstream of the transcriptional start site (TSS) are characterized by low transcriptional plasticity, while genes lacking an NFR immediately upstream of the TSS are characterized by high transcriptional plasticity. It is interesting to know whether these two classes of genes differ in their overlap percentage. The lists of genes containing and lacking an NFR were both downloaded from Tirosh and Baikai's study [26]. As shown in Figure 5a, genes containing an NFR show significantly lower overlap percentage (calculated using Equation (3)) compared with genes lacking an NFR, suggesting that containing an NFR immediately upstream of the TSS is associated with a gene being insensitive to the knockout of its promoter-binding TFs.

It is known that genes lacking an NFR are subjected to greater regulation by specific chromatin remodelling factors than are genes containing an NFR [26]. If our finding is biologically meaningful, we expect that TFs involved in chromatin remodelling have higher overlap percentage compared with the rest of the 173 TFs under study. To test this assertion, we downloaded the list of TFs involved in chromatin remodelling from Ozonov and van Nimwegen's study [27]. As expected, TFs involved in chromatin remodelling show significantly higher overlap percentage (calculated using Equation (2)) compared with the rest of the 173 TFs under study (see Figure 5b), thus further strengthen our finding.

### Genes with low transcriptional plasticity have lower overlap percentage

We have shown that two classes of genes (TATA box-less genes and genes containing an NFR) have lower overlap percentage. Since both classes of genes are known to have low transcriptional plasticity [21,26], this prompts us to speculate that genes with low transcriptional plasticity have lower percentage of the TF binding dataset overlapped with the TF knockout effect dataset than do genes with high transcriptional plasticity. To test our speculation, let us define two sets of genes. The first is the set of genes with low transcriptional plasticity, which is defined as those genes whose transcriptional plasticity are among the bottom X% of the 4065 genes under study. The other is the set of genes with high transcriptional plasticity, which is defined as those genes whose transcriptional plasticity are among the top X% of the 4065 genes under study (see Additional file 2 for details). The transcriptional plasticity each gene in the yeast genome was downloaded from Lin et al.'s study [22]. As shown in Figure 5c, genes with low transcriptional plasticity show significantly lower overlap percentage (calculated using Equation (3)) than do genes with high transcriptional plasticity, suggesting that low transcriptional plasticity is associated with a gene being insensitive to the knockout of its promoter-binding TFs. Note that our result is robust against different choices (10, 20, 30, 40 or 50) of X being used.

### Several gene properties are not associated with the overlap percentage

In the previous sections, we show that four gene properties (expression level, TATA box, nucleosome, and transcriptional plasticity) are associated with the overlap percentage. Actually, five other gene properties are also tested but do not have statistically significant association with the overlap percentage. These five gene properties include the 5'UTR length, 3'UTR length, gene essentiality, number of physical interaction partners and number of genetic interaction partners.

### More analyses motivated by Cusanovich et al's study

In Cusanovich et al.'s paper [29], they reported that functional TF binding is enriched in the regulatory regions with a larger number of bound TFs and more binding sites. Moreover, functional TF binding tends to occur further from the TSS (i.e. in the enhancer regions). Motivated by their findings, we perform extra analyses and have the following three observations: (i) a low number of bound TFs in a gene, (ii) a low number of TFBSs in a gene, and (iii) a short average distance of TFBSs to the TSS in a gene are all associated with a gene being insensitive to the knockout of its promoter-binding TFs (see Additional file 3 for details).

## Conclusions

This study gives a plausible biological explanation of a counterintuitive phenomenon: most binding targets of a yeast transcription factor are not affected when the transcription factor is knocked out. Our analyses find that TFs with high functional redundancy show significantly lower percentage than do TFs with low functional redundancy. This suggests that functional redundancy may lead to one TF compensating for another, thus masking the TF knockout effect on the binding targets of the knocked-out TF. In addition, identifying biological features that are associated with the overlap percentage may provide clues for finding other biological explanations. We show that seven gene properties (low expression level, lacking a TATA box, containing a nucleosome-free region immediately upstream of the transcriptional start site, low transcriptional plasticity, a low number of bound TFs, a low number of TFBSs, and a short average distance of TFBSs to the TSS) are associated with a gene being insensitive to the knockout of its promoter-binding TFs.

## Competing interests

The authors declare that they have no competing interests.

## Authors' contributions

WSW conceived the research topic and provided essential guidance. WSW and FJL developed the method and wrote the manuscript. FJL did all the simulations. Both authors read, edited and approved the final manuscript.

## Supplementary Material

Additional file 1 **The details of the 4065 genes under study**. For each of the 4065 genes under study, its T_B_(g), T_K_(g) and T_B_(g)∩T_K_(g) are provided. Click here for file

Additional file 2 **The details of the top/bottom X% of the expression level and transcriptional plasticity**. For each top/bottom X% case, their gene names, T_B_(g), T_K_(g), and T_B_(g)∩T_K_(g) are provided. Click here for file

Additional file 3 **The details of the analyses motivated by Cusanovich et al's study**. Click here for file

## Declarations

The publication of this paper was funded by National Cheng Kung University and Ministry of Science and Technology of Taiwan MOST-103-2221-E-006 -174 -MY2.

This article has been published as part of *BMC Systems Biology *Volume 9 Supplement 6, 2015: Joint 26th Genome Informatics Workshop and 14th International Conference on Bioinformatics: Systems biology. The full contents of the supplement are available online at http://www.biomedcentral.com/bmcsystbiol/supplements/9/S6.
