# Supplementary material for: Functional redundancy of transcription factors explains why most binding targets of a transcription factor are not affected when the transcription factor is knocked out
Source: BMC Syst Biol. 2015 Dec 9;9(Suppl 6):S2. doi: 10.1186/1752-0509-9-S6-S2 (PMC4674858; doi:10.1186/1752-0509-9-S6-S2)
Supplement: Additional file 3 — The details of the analyses motivated by Cusanovich et al's study. [file 1752-0509-9-S6-S2-S3.docx]

## More analyses motivated by Cusanovich et al’s study

In Cusanovich et al.’s paper [1], they reported that functional TF binding is enriched in the regulatory regions with a larger number of bound TFs and more binding sites. Moreover, functional TF binding tends to occur further from the TSS (i.e. in the enhancer regions). Motivated by their findings, we perform the following three analyses.

First, we test whether a low number of TFs that bind to a gene is associated with a gene being insensitive to the knockout of its promoter-binding TFs. To do the testing, let us define two sets of genes. The first is the set of genes which are bound by a low number of TFs, defined as those genes whose bound TF numbers are among the bottom X% (X=10, 20, 30, 40 or 50) of the 4065 genes under study. The other is the set of genes which are bound by a high number of TFs, defined as those genes whose bound TF numbers are among the top X% (X=10, 20, 30, 40 or 50) of the 4065 genes under study. Note that the number of TFs that bind to a gene is defined using the TF binding site (TFBS) data from MacIsaac et al.’s study [2]. As shown in Figure 1, the set of genes which are bound by a low number of TFs show significantly lower overlap percentage compared with the set of genes which are bound by a high number of TFs, suggesting that the low number of TFs that bind to a gene is associated with a gene being insensitive to the knockout of its promoter-binding TFs. Note that our result is robust against different choices (10, 20, 30, 40 or 50) of X and different qualities of MacIsaac et al’s TFBS data ((a) nobindc1 TFBS data or (b) p005c1 TFBS data) being used.

## Figure 1


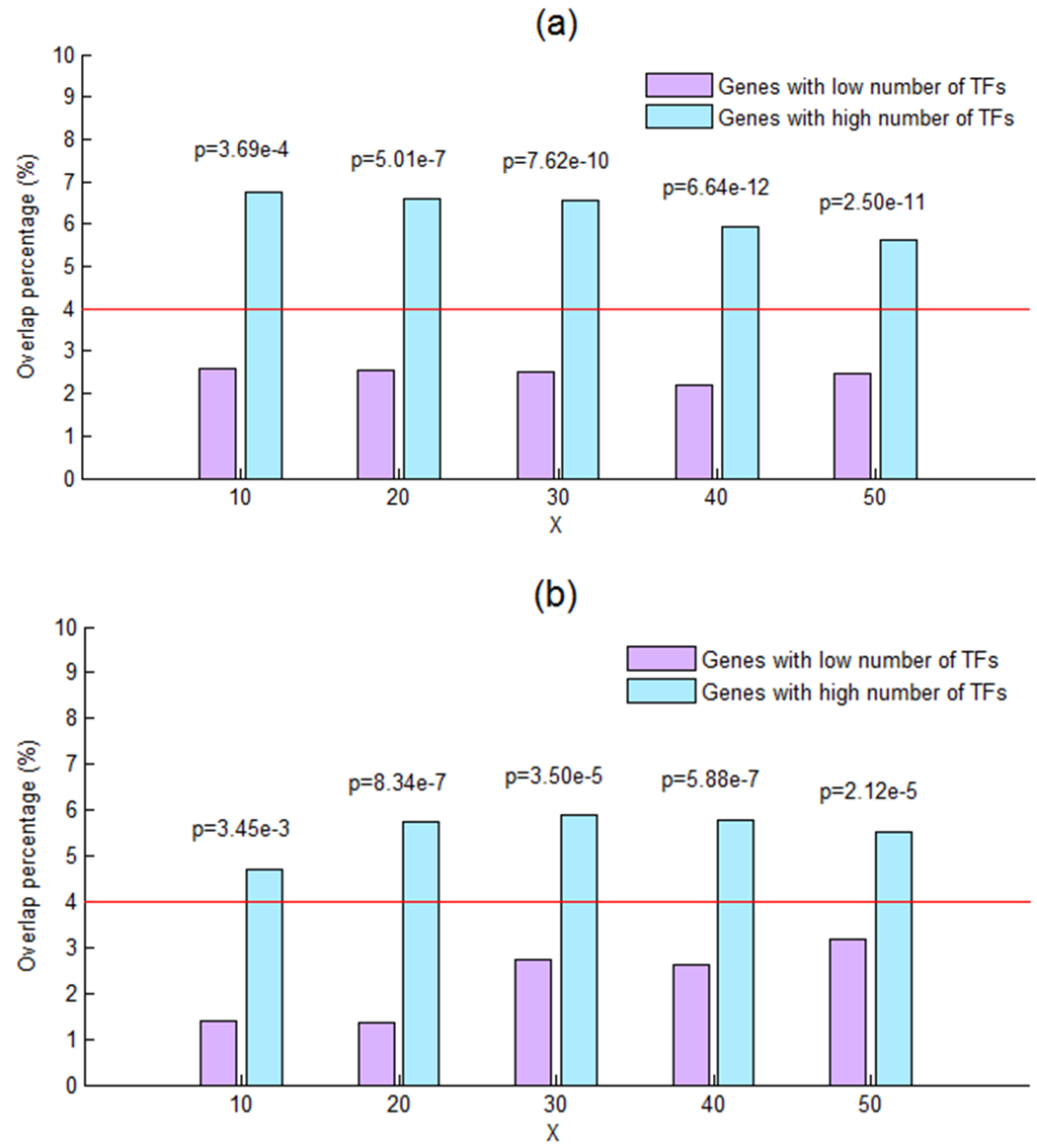


Second, we test whether a low number of TFBSs in a gene is associated with a gene being insensitive to the knockout of its promoter-binding TFs. To do the testing, let us define two sets of genes. The first is the set of genes which have a low number of TFBSs, defined as those genes whose total TFBS numbers are among the bottom X% (X=10, 20, 30, 40 or 50) of the 4065 genes under study. The other is the set of genes which have a high number of TFBSs, defined as those genes whose total TFBS numbers are among the top X% (X=10, 20, 30, 40 or 50) of the 4065 genes under study. Note that the number of TFBSs in a gene is defined using the TFBS data from MacIsaac et al.’s study [2]. As shown in Figure 2, the set of genes which have a low number of TFBSs show significantly lower overlap percentage compared with the set of genes which have a high number of TFBSs, suggesting that a low number of TFBSs in a gene is associated with a gene being insensitive to the knockout of its promoter-binding TFs. Note that our result is robust against different choices (10, 20, 30, 40 or 50) of X and different qualities of MacIsaac et al’s TFBS data ((a) nobindc1 TFBS data or (b) p005c1 TFBS data) being used.

## Figure 2


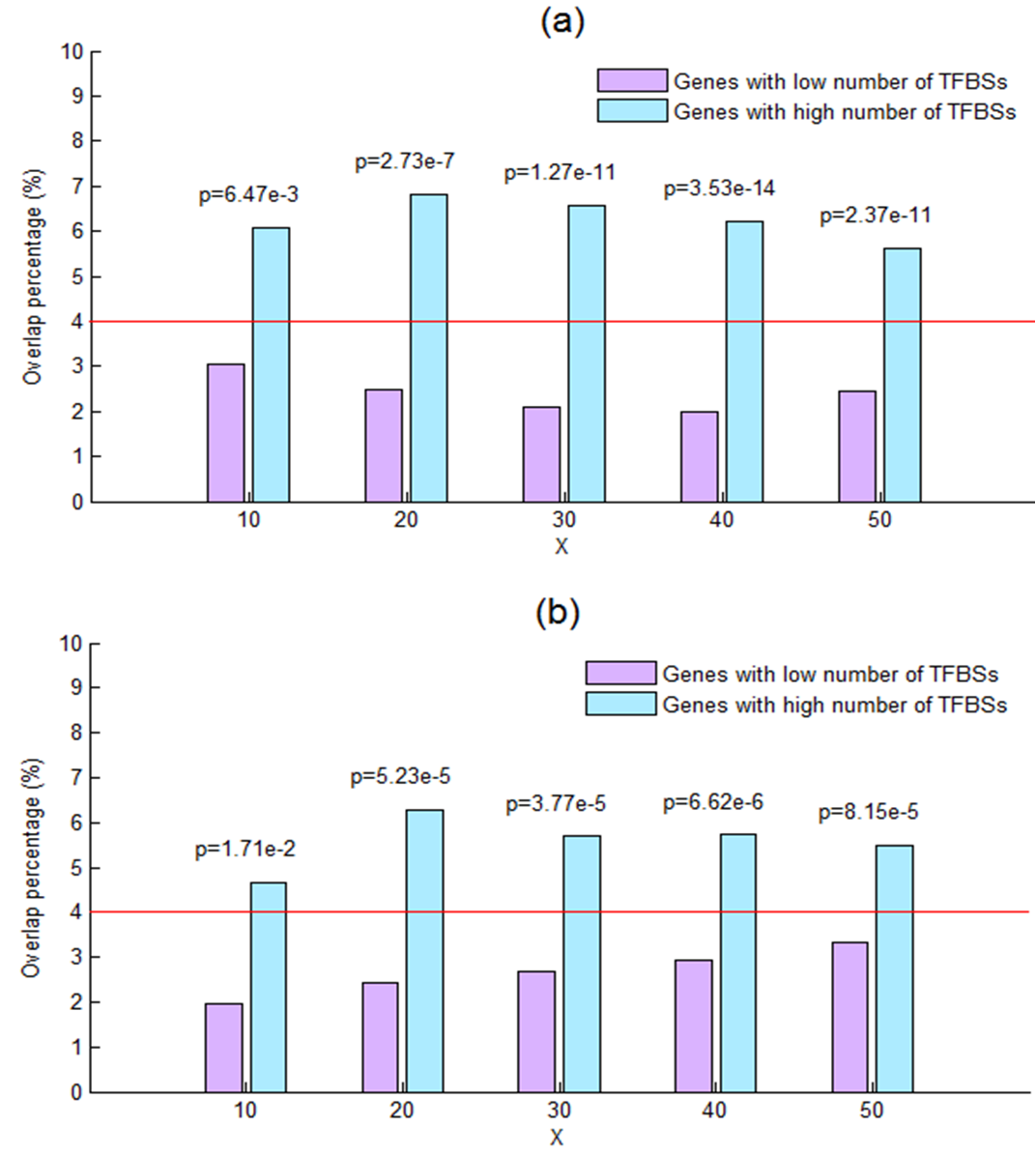


Third, we test whether the short average distance of TFBSs from the TSS in a gene is associated with a gene being insensitive to the knockout of its promoter-binding TFs. To do the testing, let us define two sets of genes. The first is the set of genes whose TFBSs have a short average distances from the TSS, defined as those genes with their TFBSs having average distances from the TSS among the bottom X% (X=10, 20, 30, 40 or 50) of the 4065 genes under study. The other is the set of genes whose TFBSs have a long average distances from the TSS, defined as those genes with their TFBSs having average distances from the TSS among the top X% (X=10, 20, 30, 40 or 50) of the 4065 genes under study. Note that the average distance of TFBSs from the TSS in a gene is defined using the TFBS data from MacIsaac et al.’s study [2]. As shown in Figure 3, the set of genes whose TFBSs have a short average distances from the TSS show significantly lower overlap percentage compared with the set of genes whose TFBSs have a long average distances from the TSS, suggesting that a short average distances of TFBSs from the TSS in a gene is associated with a gene being insensitive to the knockout of its promoter-binding TFs. Note that our result is robust against different choices (10, 20, 30, 40 or 50) of X and different qualities of MacIsaac et al’s TFBS data ((a) nobindc1 TFBS data or (b) p005c1 TFBS data) being used.

## Figure 3


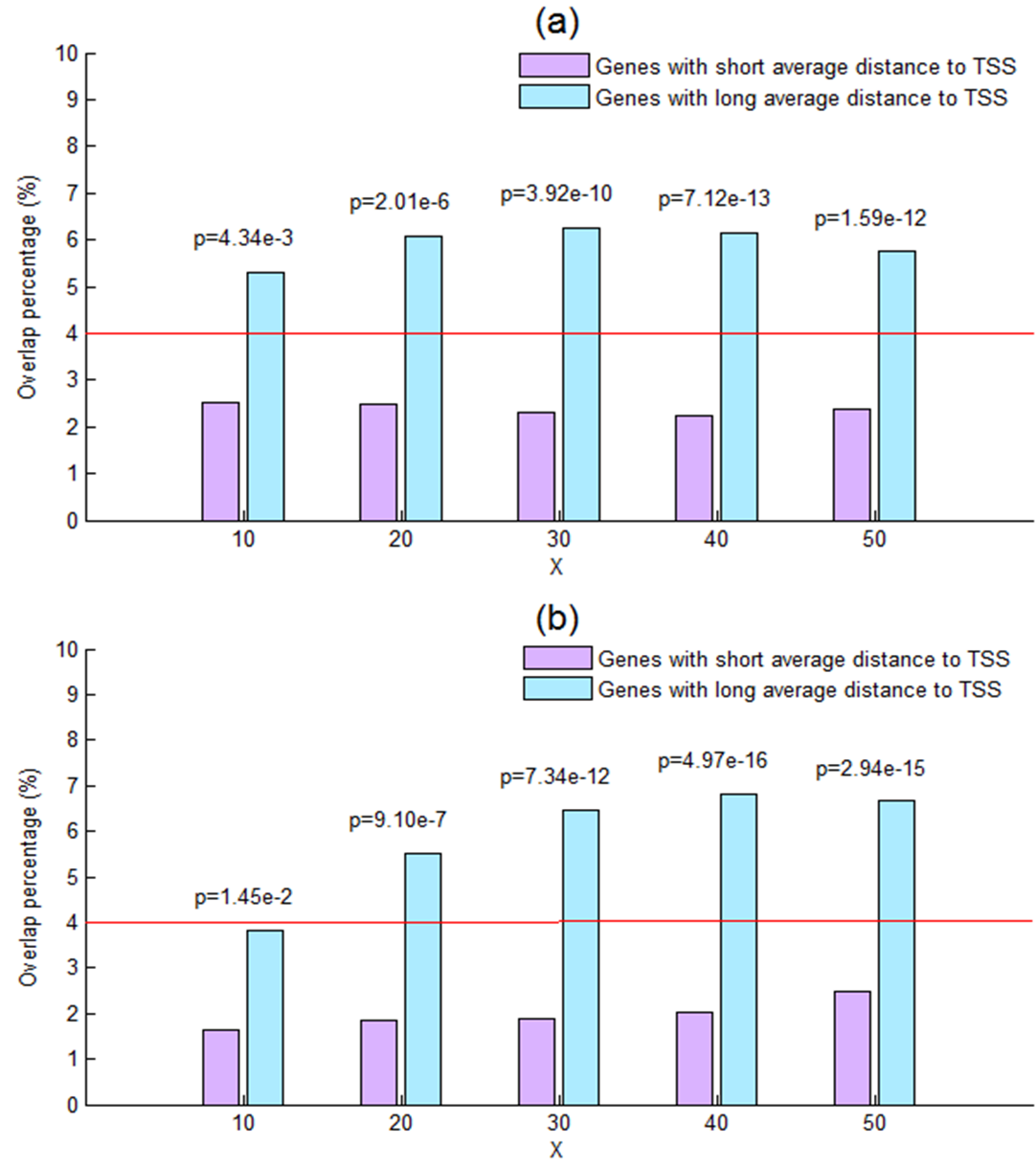


# References

1. Cusanovich DA, Pavlovic B, Pritchard JK, Gilad Y: **The functional consequences of variation in transcription factor binding.** *PLoS Genet* 2014, **10(3)**:e1004226.
2. MacIsaac KD, Wang T, Gordon DB, Gifford DK, Stormo GD, Fraenkel E: **An improved map of conserved regulatory sites for Saccharomyces cerevisiae.** *BMC Bioinformatics* 2006, **7:**113.
